# Development and validation of a protocol for documentation of obstetric perineal lacerations

**DOI:** 10.1007/s00192-019-03915-y

**Published:** 2019-03-19

**Authors:** Markus Harry Jansson, Kerstin Nilsson, Karin Franzén

**Affiliations:** 1 Department of Obstetrics and Gynecology, Örebro University Hospital, SE-701 85 Örebro, Sweden; 2 School of Medical Sciences, Faculty of Health and Medicine, Örebro University, Örebro, SE-701 82 Sweden

**Keywords:** Health administrative data, Obstetric anal sphincter injuries, Perineal tear, Validation studies

## Abstract

**Introduction and hypothesis:**

The aim of this study was to develop a new protocol for documentation of perineal lacerations and to validate the latter against the most common obstetric record system in Sweden. The hypothesis was that the new protocol would render more complete data on perineal lacerations than the current documentation method.

**Methods:**

A protocol for documentation of perineal lacerations was developed to be sufficiently comprehensive to serve research purposes. All women delivering their first child vaginally from 13 October 2015 to 1 February 2016 at Örebro University Hospital were eligible for the validation study. Perineal lacerations were documented using the protocol in parallel with the regular obstetric record system (ObstetriX). Cross tabulations were used to compare the coverage regarding perineal lacerations between the two documentation methods. McNemar’s test was used to evaluate systematic differences between the methods.

**Results:**

A total of 187 women were included. The coverage of documentation regarding perineal laceration was significantly higher (*p* < 0.001) in the new protocol (89%) compared with ObstetriX (18%). Incidence of second-degree perineal tears was 26% according to the new protocol and 11% according to ObstetriX. The incidence of third-degree perineal tears A, B, and C was 2.7%, 2.1%, and 2.1%, respectively, according to the new protocol, and 3.2%, 2.7%, and 1.1% according to ObstetriX.

**Conclusions:**

This validation study of a new documentation protocol showed that it delivered significantly more comprehensive information regarding perineal lacerations than the most common obstetric record system in Sweden.

**Electronic supplementary material:**

The online version of this article (10.1007/s00192-019-03915-y) contains supplementary material, which is available to authorized users.

## Introduction

During vaginal delivery about 80% of women contract some degree of perineal trauma, primiparous women more frequently than multiparous women [1, 2]. The majority of tears are first- or second-degree perineal tears, but third- and fourth-degree tears occur in 3.2–4.3% of vaginal deliveries irrespective of parity [1, 2]. In Sweden during 2009–2013, 6.2% of primiparas contracted a third- or fourth-degree tear [3]. Obstetric anal sphincter injuries are the largest risk factor for developing anal incontinence among women [4], so these perineal tears merit attention. Second-degree perineal lacerations have been suggested as a “marker” for perineal trauma sufficient to cause anal sphincter injury, meaning that women with these tears should be thoroughly examined [5]. The clinical diagnosis of obstetric and sphincter injuries has proven difficult [5]. Endoanal ultrasound performed at the delivery ward diagnoses obstetric anal sphincter defects more accurately than a clinical examination [5], but has not yet become a routine method.

Administrative discharge codes are increasingly used in health services research, both as a measure of quality of care and for scientific purposes. In Sweden, national monitoring of perineal tears and other obstetric complications is pursued through the Pregnancy Registry (Graviditetsregistret). Data are automatically extracted from the medical record system in most Swedish counties. The validity of the scientific studies and national quality monitoring based on discharge codes heavily depends on the accuracy of the available data. Two previous American studies on the accuracy of third- and fourth-degree perineal tears have shown varying results. Romano et al. [6] showed accurately reported diagnoses, while Brubaker et al. [7] found that nearly a quarter of hospital discharges associated with a third- or fourth-degree tear were undercoded. Thus, before drawing too far-reaching conclusions from comparisons of quality of care, the validity of the diagnostic codes must be established.

Standardized documentation is crucial to achieve robust data, both for national monitoring of quality of care and for scientific studies. A PubMed search revealed only one protocol [1] for documentation of perineal lacerations; however, this protocol constructed by Samuelsson et al was neither adapted to the most recent ICD classification [8] nor validated. The aim of this study was to develop a new protocol for documentation of perineal lacerations and to validate the latter against the most common obstetric record system in Sweden. The hypothesis of this study was that the new protocol would give more detailed information about the degree of perineal tears and suturing for individual women than the present obstetric record system, ObstetriX. We also hypothesized that the coverage of women being comprehensively documented regarding their perineal tears and suturing would increase when using the new documentation protocol.

## Materials and methods

A protocol for documentation of perineal lacerations and suturing after vaginal delivery was developed (see Figure S1 in electronic supplementary material). The protocol contains information about perineal trauma as a risk factor for future pelvic floor symptoms, including degree of perineal tear, episiotomy, anterior lacerations and labial lacerations, and specific information regarding suturing of the different lacerations. The protocol was developed to fulfill the needs of a future cohort study of pelvic floor dysfunction in women delivering their first child. The protocol uses the Royal College of Obstetricians and Gynecologists classification of perineal lacerations [8], and the distinction between low and high obstetric vaginal laceration is based on the ICD-10 classification [9]. Regarding other obstetric vulvar trauma, we used our own clinical experience when developing the questions in the protocol. We aimed to construct a legible protocol configured as a checklist in order to minimize loss of information. A group of midwives and obstetricians at our Department of Obstetrics and Gynecology reviewed the protocol and gave feedback regarding the content and design of the protocol.

All women delivering their first child vaginally from 13 October 2015 to 1 February 2016 at Örebro University Hospital were eligible for the study. The women were included consecutively. The approximate sample size required was estimated based on clinical and scientific experience. No formal power calculation was pursued when planning the study. After delivery the midwife or, where appropriate, the obstetrician in charge documented the perineal laceration and suturing both in the protocol mentioned above and simultaneously in the regular computerized obstetric record system (ObstetriX, Siemens, version 2.14.02.200). In ObstetriX, the midwife documents perineal lacerations and vaginal ruptures in the computerized sheet “Delivery care 1” (Förlossningsvård 1, see Table S1 in electronic supplementary material). In the case of a more extensive perineal tear or vaginal rupture that is sutured by an obstetrician, the obstetrician will document the injury and the suturing in the protocol “Suturing of delivery-related injury” (Sutur av förlossningsskada; see Table S2 in electronic supplementary material). When finishing the documentation regarding a third- or fourth-degree perineal tear, a pop-up window appears with a list and descriptions of the diagnostic codes of third- and fourth-degree perineal tears according to ICD-10, allowing the obstetrician to choose one. The ICD-10 diagnostic codes correspond to the Royal College of Obstetricians and Gynecologists’ classification of perineal tears [8]. In the present study, the main author extracted data from the medical record using an accessory program called ObstetriX Delivery Ward Ledger (ObstetriX Förlossningsliggare, version 2.14.02.200, Siemens). To achieve the subclassification of third-degree perineal tears into A, B, and C, the diagnostic code was extracted manually from the medical record.

### Statistical analysis

Continuous data were summarized as means and standard deviations and categorical data as percentages. Cross tabulation was used to compare the coverage of documentation regarding the degree of perineal tear between ObstetriX and the new protocol. Percentage of women in agreement and kappa were used to estimate the concordance and McNemar’s test to evaluate systematic differences between the methods regarding labial tears, vaginal ruptures, and episiotomy. The size of the estimated kappa was judged according to accepted statistical standards [10]: < 0.20 poor, 0.21–0.40 fair, 0.41–0.60 moderate, 0.61–0.80 good, and 0.81–1.00 very good. Version 22 of the SPSS software package (IBM Corp., Armonk, NY) was used, and the confidence intervals for percentage agreement and kappa were calculated using VassarStats: Website for Statistical Computation (http://vassarstats.net/).

Ethical approval was given by the Regional Ethical Review Board in Uppsala (registration no. 2015/318).

## Results

During the study period, 310 women delivered their first child at the Örebro University Hospital. Of these, 40 women (13%) delivered by cesarean section and were not eligible. Thus, 270 women delivered vaginally and were eligible for the study; 187 of them were included. The reason for the non-inclusion of the remaining 87 women was likely a lack of awareness about the study among the midwives and doctors, meaning that the women were not asked for their consent to participate. The distribution between spontaneous and instrumental delivery among study participants was 160 (86%) and 27 (14%), respectively. Patient and delivery characteristics of study participants are presented in Table 1.

**Table 1:** Patient and delivery characteristics of study participants

	Mean	Range	Standard deviation	Missing data (number of patients)
Age (years)	27.3	18–41	4.8	0
Maternal weight^a^ (kg)	68.1	44–123	14.0	10
Maternal height (cm)	165.2	145–183	6.6	8
Maternal BMI (kg/m^2^)	25.0	18.1–44.1	4.9	10
Gestational age at delivery	39 weeks + 6 days	30 weeks + 2 days–42 weeks + 2 days	1 week + 5 days	0
Birth weight (g)	3443	3080–4760	517	0
Height at birth (cm)	50	41–56	2.4	0
Head circumference at birth (cm)	34.7	29–39	1.5	0

^a^Maternal weight at registration to maternal health care in early pregnancy

### Perineal tears

The coverage of documentation of perineal lacerations in ObstetriX and the new documentation protocol is presented in Table 2. For the new documentation protocol, coverage of documentation was taken to correspond to the rate of comprehensively filling in the protocol regarding perineal tear (see question 7 in Figure S1 in electronic supplementary material), which meant ticking one of the “Yes” or “No” boxes under the heading “Perineal tear” and choosing a degree when a perineal tear was present. For ObstetriX, coverage information was derived from the documentation protocol “Suturing of delivery-related injury” (see Table S1 in electronic supplementary material). The new documentation protocol gave comprehensive information about the existence or absence of perineal lacerations in 89% of women, while in ObstetriX the corresponding information was available only in 18% of women (*p* < 0.001).

**Table 2:** Contingency table showing coverage of documentation of perineal laceration in ObstetriX and in the new documentation protocol

		New documentation protocol		Total	%	McNemar
ObstetriX		Information available	No information available			Level of significance (2-sided)
	Information available	33	1	34	18%	*p* < 0.001
	No information available	134	19	153	11%	
	Total	167	20	187		
	%	89%	11%			

The incidences of different degrees of perineal tears according to ObstetriX and the new protocol are presented as a contingency table (see Table 3). There was no information about first-degree perineal tears in ObstetriX. The incidence of second-degree perineal tears was 26% according to the new documentation protocol and 11% according to ObstetriX.

**Table 3:** Contingency table showing the incidence of different degrees of perineal tears according to ObstetriX and the new documentation protocol

			New documentation protocol								
		1°	2°	3°A	3°B	3 °C	No perineal laceration	No specification of degree of perineal laceration	N/A	Total	%
	2°	1	19	0	0	0	0	1	0	21	11%
	3°A	0	0	5	0	1	0	0	0	6	3.2%
ObstetriX	3°B	0	0	0	4	0	1	0	0	5	2.7%
	3 °C	0	0	0	0	2	0	0	0	2	1.1%
	3°, not specified	0	0	0	0	1	0	0	0	1	0.5%
	N/A	34	31	0	0	0	68	0	19	152	81%
	Total	35	50	5	4	4	69	1	19	187	
	%	19%	26%	2.7%	2.1%	2.1%	37%	0.5%	10%		

N/A, no information available

The information obtained regarding third-degree perineal tears in ObstetriX and in the new documentation protocol is presented in Table 4. The diagnoses agreed in all but 3 of the 14 women affected. In woman no. 5, the diagnosis was more severe according to the new documentation protocol compared with ObstetriX. In woman no. 184, a diagnosis was missing in the new documentation protocol, and in woman no. 155, there was no specification about the extension of the laceration in ObstetriX. The only information on suturing that can be extracted from ObstetriX is the method (that is, end-to-end or overlap); no distinction is made regarding suturing technique in the external and internal anal sphincter, respectively. The new documentation protocol gave comprehensive information about suturing methods of both the external and internal anal sphincter, including the suturing material and number of sutures in the internal anal sphincter; however, information on the number of sutures in the external sphincter was only present for 5 of 13 women.

**Table 4:** Information obtained regarding third-degree perineal tears in ObstetriX and the new documentation protocol

Patient ID	Degree of perineal tear								
	ObX	NP	Suturing of 3rd-degree tear Obx	Suturing method of EAS NP	Suturing method of IAS NP	Number of sutures in EAS NP	Suturing material EAS NP	Number of sutures IAS NP	Suturing material IAS NP
5	3A	3C	End-to-end	–	Simple	–	–	2	Polysorb
34	3A	3A	End-to-end	End-to-end	–	N/A	Polysorb	–	–
44	3A	3A	End-to-end	End-to-end	–	N/A	Polysorb	–	–
47	3A	3A	End-to-end	End-to-end	–	2	Polysorb	–	–
64	3A	3A	End-to-end	End-to-end	–	N/A	PDS	–	–
185	3A	3A	End-to-end	End-to-end	–	N/A	Polysorb	–	–
67	3B	3B	End-to-end	End-to-end	–	N/A	Polysorb	–	–
76	3B	3B	End-to-end	End-to-end	–	3	Polysorb	–	–
84	3B	3B	Overlap	Overlap	–	2	Polysorb	–	–
176	3B	3B	End-to-end	End-to-end	–	N/A	Polysorb	–	–
184	3B	N/A	End-to-end	End-to-end	–	2	Polysorb	–	–
59	3C	3C	End-to-end	End-to-end	Simple	N/A	Vicryl	N/A	Vicryl
135	3C	3C	End-to-end	End-to-end	Simple	1	N/A	3	N/A
155	3X^a^	3C	N/A	N/A	Simple	N/A	N/A	2	Polysorb

If information is missing where it is expected to be found, it is described as not available (N/A). If information is not expected, it is noted as "–" where it is absent. ^a^3X refers to a third-degree perineal tear without specification

Obx, ObstetriX; NP, new protocol regarding perineal laceration and suturing; N/A, not available; EAS, external anal sphincter; IAS, internal anal sphincter

### Other vulvar or vaginal trauma related to delivery

Agreement between ObstetriX and the new documentation protocol regarding the variables labial laceration, vaginal rupture, and episiotomy is given here in contingency tables (see Tables 5, 6, and 7). The tables also show results of McNemar’s test, agreement, and kappa value for each of the variables, respectively. Information was missing in the new documentation protocol for two women regarding labial tears, for seven women regarding vaginal rupture, and for two women regarding episiotomy. In ObstetriX, it was impossible to tell if information was missing regarding these parameters, since absence of information could mean either no lesion or no information.

**Table 5:** Contingency table showing the absence and incidence of labial tear according to ObstetriX and the new documentation protocol, respectively

	Labial tear NP^a^	No labial tear NP^a^	Total	%	Agreement: (95% CI)	McNemar
Labial tear ObX	78	15	93	50%	0.86 (0.80–0.90)	Exact significance (2-sided)
No labial tear ObX	11	81	92	50%	Kappa: (95% CI)	*p = *0.557^b^
Total	89	96	185		0.72 (0.62–0.82)	
%	48%	52%				

Information was missing regarding two women, hence the total number of 185 women

^a^In the new documentation protocol labial tears are specified as those requiring suturing, which is not the case in Obstetrix. ^b^Calculated using binominal distribution. ObX, ObstetriX. NP, the new protocol for documentation of perineal lacerations

**Table 6:** Contingency table showing the absence and incidence of vaginal rupture according to ObstetriX and the new documentation protocol, respectively

	Vaginal rupture NP	No vaginal rupture NP	Total	%	Agreement: (95% CI)	McNemar
Vaginal rupture ObX	133	2	135	75%	0.88 (0.82–0.92)	Exact significance (2-sided)
No vaginal rupture ObX	20	25	45	25%	Kappa: (95% CI)	*p* = 0.001^a^
Total	153	27	180		0.62 (0.48–0.77)	
%	85%	15%				

Information was missing for seven women, hence the total number of 180 women

^a^Calculated using binominal distribution. ObX, ObstetriX. NP, the new protocol for documentation of perineal lacerations

**Table 7:** Contingency table showing the absence and presence of episiotomy according to ObstetriX and the new documentation protocol, respectively

	Episiotomy NP	No episiotomy NP	Total	%	Agreement: (95% CI)	McNemar
Episiotomy ObX	12	0	12	6%	0.95 (0.91–0.97)	Exact significance (2-sided)
No episiotomy ObX	9	164	173	94%	Kappa: (95% CI)	*p =* 0.04^a^
Total	21	164	185		0.70 (0.51–0.89)	
%	11%	89%				

Information was missing for two women, hence the total number of 185 women

^a^Calculated using binominal distribution. ObX, ObstetriX. NP, the new protocol for documentation of perineal lacerations

The proportion of women contracting labial tears was almost identical between ObstetriX and the new documentation protocol. However, 11 women had a labial tear according to the new protocol but not according to ObstetriX, and 15 women had a labial tear according to ObstetriX but not according to the new protocol. For measuring concordance, the percentage in agreement was 0.86 and kappa was 0.72; the latter is interpreted as good agreement according to the previously cited standard work [8]. The new protocol showed a significantly higher proportion of women sustaining a vaginal rupture compared with ObstetriX (85% versus 75%; *p* = 0.001). There were very few cases where vaginal rupture was documented in ObstetriX and not in the new documentation protocol, but the opposite situation was seen in several cases. The percentage in agreement was 0.88, and kappa was 0.62, which is classified in the lower range of good agreement. Episiotomies were documented to a greater extent in the new documentation protocol than in ObstetriX (11% versus 6%; *p* = 0.04). No case was found where episiotomy was documented in ObstetriX but not in the new documentation protocol. Conversely, almost half of the women with an episiotomy documented in the new protocol were not similarly documented in ObstetriX. The percentage in agreement was 0.95, and kappa was 0.70, which is classified as good agreement.

## Discussion

The present study shows that the new documentation protocol delivers more comprehensive information regarding perineal lacerations compared with the current obstetric record system, ObstetriX, when used in primiparous women. The new protocol appears to specifically diagnose second-degree perineal lacerations to a greater extent than ObstetriX, which implies that the incidence of these perineal lacerations might be systematically underestimated. We could not see any difference in the ability to diagnose third-degree perineal tears, but the new protocol gave more comprehensive information regarding suturing. The agreement between the new protocol and ObstetriX was generally good when comparing labial lacerations, vaginal ruptures, and episiotomies.

### Strengths and limitations

To our knowledge, this is the first validation of a protocol for documentation of perineal lacerations. The lack of a previously validated protocol implies that we do not know the true incidence of perineal lacerations when comparing the two documentation methods, which is a limitation. The index method used in this study, ObstetriX, is far from suitable as a reference method for documentation of perineal trauma because of the substantial lack of information. Many of the estimates of diagnostic accuracy suggested for use in validation of health administrative data, such as sensitivity and specificity [11], were thus considered inappropriate to use. The ideal reference method would be clinical examination by experts in perineal rupture classification, but this method was not practically possible to implement. Due to this lack of a previously validated protocol, validation against the current obstetric medical record system in Sweden was considered acceptable. Since the Royal College of Obstetricians and Gynecologists classification of perineal rupture was used, the results may be transferred to an international context.

We studied the documentation for 187 women, which is a sufficient number to make a statistical comparison of the documentation methods except for third- and fourth-degree perineal tears, where the numbers were too small to compare with statistical methods. The fact that no power calculation was made when planning the study is a limitation. A considerably larger study sample would be required to achieve the power to compare the documentation methods regarding third- and fourth-degree perineal tears, but that was judged not feasible within a reasonable period of time. The study only includes one obstetric unit, which possibly limits the external validity; however, we have no specific reason to believe that the result would change if we repeated the study in another obstetric unit. The fact that documentation was made by the same person in both ObstetriX and the new documentation protocol for each woman indicates that the differences observed are due to the different configurations of the protocols and are not random.

There was a loss of 87 women who were eligible for the study but for unknown reasons were not included. It is possible that in many cases the reason for non-inclusion was that the midwife or obstetrician was not aware of the study; however, it is hard to completely rule out any form of selection bias.

We found that the new documentation protocol had better coverage in terms of information on perineal lacerations. This might because this protocol specifically asks if there was a perineal laceration and, if so, what degree of perineal laceration there was. Conversely, in ObstetriX, accessible information about the degree of perineal laceration is only generated if the documentation protocol “Suturing of delivery-related injury” is used, which was only the case if the laceration was sutured by an obstetrician rather than a midwife. Since the majority of second-degree perineal lacerations are sutured by midwives, no diagnosis is registered in those cases. The documentation protocol of ObstetriX only includes classification into second-, third-, and fourth-degree perineal tears, but not first-degree perineal tear, so there is no information about these lacerations in ObstetriX.

The new documentation protocol appears to diagnose second-degree perineal tears to a greater extent than ObstetriX. The current Swedish documentation method might systematically underestimate second-degree perineal lacerations, which is unfortunate given the increasing research interest in these lacerations. Second-degree perineal lacerations might cause future morbidity if not sutured correctly or if complicated, for example, by an infection. Additionally, second-degree perineal tears might hide an occult sphincter rupture, which is another reason why these lacerations merit further attention [4]. Overall, the two documentation methods appear to have acceptable agreement on diagnosis of third-degree perineal lacerations; however, we refrained from calculating agreement and testing for statistically significant differences regarding these lacerations, as the numbers of cases were too small.

Overall, the agreement is good between the two documentation methods regarding labial tears, vaginal rupture, and episiotomy. The two documentation methods seem to give equally comprehensive information about labial lacerations. In the documentation protocol, labial tears are specified as those requiring suturing, which is not the case in ObstetriX. To some extent, this might explain the cases where ObstetriX and the new documentation protocol disagree. In the case of vaginal rupture and episiotomy, the new documentation protocol gives more information than ObstetriX. This may be because the new protocol focuses more specifically on the perineal and vaginal trauma that has occurred compared with the protocol “Delivery care 1” in ObstetriX.

One could expect that the information obtained from a documentation protocol that asks specific and detailed questions about obstetric perineal trauma would be even more complete than this study showed. The fact that the new documentation protocol was filled in on a physical sheet of paper might partly explain this. A computerized documentation protocol with mandatory questions that cannot be signed before all necessary tick boxes are filled in would increase coverage of information even more.

To our knowledge, a validated protocol for documentation of obstetric perineal laceration in a research context has been lacking. In the present study, we have developed and validated a new documentation protocol which has proved to be suitable for scientific purposes. The current Swedish documentation method, on the other hand, appears to systematically underestimate the incidence of second-degree perineal tears. The increasing demand for health care organizations to follow up quality of care makes this new protocol useful to implement in future obstetric record systems.

## Electronic supplementary material

ESM 1 (DOCX 26 kb)
