# Supplementary material for: Development and validation of a protocol for documentation of obstetric perineal lacerations
Source: Int Urogynecol J. 2019 Mar 19;30(12):2069–76. doi: 10.1007/s00192-019-03915-y (PMC6861348; doi:10.1007/s00192-019-03915-y)
Supplement: Supplementary file 1 — (DOCX 26 kb) [file 192_2019_3915_MOESM1_ESM.docx]

Electronic supplementary material

Figure S1. A protocol for documentation of perineal lacerations and suturing after vaginal delivery. The original version of the protocol used in the study was written in Swedish. The version of the protocol below has been translated to English. The original Swedish version of the protocol can be provided on request.

**Protocol for documentation of perineal tears**

**Date of delivery:** ______________________

**Patient’s name:** __________________________

**Patient’s personal identity number:** __________________

| Has the woman been subject to female mutilation? | Yes  No  if Yes → | Was there need for deinfibulation?  Yes  No  if Yes → It was sutured,  suture  material→  Left without  suturing | | Vicryl rapid  Monocryl  Other: |  |
| --- | --- | --- | --- | --- | --- |
| Anterior lacerations  (close to clitoris or urethra, not related to female mutilations) | Yes  No  if Yes → | It was sutured, suture  material →  Left without suturing | | Vicryl rapid  Monocryl  Other: |  |
| Was episiotomy performed^a^? | Yes  No  if Yes → | Left-sided  Right-sided  Suture material → | | Polysorb  Vicryl rapid  Monocryl  Other: |  |
| Was there any labial laceration that was sutured? | Yes  No  if Yes → | Suture material → | | Vicryl rapid  Monocryl  Other: |  |
| Vaginal lacerations | Yes  No  if Yes → | Only the distal third of the  vagina was engaged  The laceration was more  extensive than the distal third  of the vagina  It was sutured, suture  material →  Left without suturing | | Polysorb  Vicryl rapid  Monocryl  Other: |  |
| Perineal body, thickness by palpation after delivery* | <1 cm  1-2 cm  > 2 cm |  | |  |  |
| Perineal tear | Yes  No |  | |  |  |
|  | if Yes** → | 1^st^ degree (vaginal epithelium or perineal skin)  2^nd^ degree (including superficial or deep  perineal muscles)  3^rd^ degree, A (rupture of < 50 % of external  anal sphincter thickness)  3^rd^ degree, B (rupture of > 50 % of external anal  sphincter thickness)  3^rd^ degree, C (rupture of internal anal sphincter)  4^th^ degree (rupture of anal epithelium)/  Laceration not possible to categorize with  involvement of anal epithelium*** | | | |
| Perineal skin | Intracutanous  suturing  Interrupted  sutures:____  Was left without  suturing | | Suture material → | Vicryl rapid  Monocryl  Other: |  |
| Perineal body | Was sutured:  Interrupted sutures  number:____  Continous suturing | | Suture material → | Polysorb  Vicryl rapid  Other: |  |
| External anal sphincter | Was sutured:  end-to-end  overlap  number:____ | | Suturmaterial → | Polysorb  PDS  Other: |  |
| Internal anal sphincter | Was sutured:  Mattress sutures  Simple sutures  Number:____ | | Suture material → | Polysorb  PDS  Other: |  |
| Anal epithelium | Was sutured:  Interrupted sutures  Number:____  Continous | | Suture material → | Polysorb  Other: |  |
| Was antibiotic prophylaxis given? | Yes  No  if Yes → | | Antibiotic prophylaxis: | Cefuroxim  Metronidazol  Other: |  |
| The laceration was assessed by: | Midwife  Physician  Both professions | |  |  |  |
| The laceration was sutured by: | Midwife  Physician  Both professions | |  |  |  |
| *The perineal body: the muscular structure between the vagina and the anal canal. The thickness is assessed by palpating with one finger in the vagina and one fingera in the anal canal simultaneously.  **In case of a perinal tear of the 3^rd^ or 4^th^ degree the protocol should be filled-in in consultation with the physician in charge.  ***For example in a so called ”buttonhole” rectal tear there is defect between the vagina and the rectum but the anal sphincter and the perineum are intact. | | | | | |
| Accessory information: | ______________________________________________________  ______________________________________________________ | | | | |

Was filled-in by:

__________________________ __________________________

Midwife Physician

^a^mediolateral episiotomy is presumed since this is the only kind of episiotomy known to be performed at the present delivery ward

Table S1. Documentation of perineal tears and suturing in “Delivery care 1” (Förlossningsvård 1) in ObstetriX.

| Item | Options |
| --- | --- |
| Episiotomy | Right, Median, Left |
| Tears | Clitoris or labia, vagina, perineum, sphincter, rectum, cervix |
| Sutures | Number of internal sutures, material of inner sutures  Number of external sutures, intracutaneous, material of external sutures |

Table S2. Documentation protocol “Suturing of delivery-related injury” (Sutur av förlossningsskada). Only the information regarding classification of perineal lacerations and vaginal ruptures and the corresponding suturing is presented.

| Item | Options |
| --- | --- |
| Vaginal rupture (possible to documents two different ruptures, at two different strokes of the clock) | Direction according to stroke of the clock (whole number)  Distal third of the vagina/All the way to fornix  Deep/Superficial  Separate sutures (number)/continuous  Suture material |
| Perineal tear | Not stated/2^nd^ degree/3^rd^ degree/4^th^ degree |
| If: 3^rd^ or 4^th^ degree tear | Total anal sphincter rupture/Partial anal sphincter rupture  If partial: <half of the anal sphincter torn/>half of the anal sphincter torn |
| If 4^th^ degree tear tear | Anal epithelium rupture length (cm)  Number of sutures/continuous  Suture material |
| Suturing of anal sphincter | Number of sutures  Not stated suturing technic/end to end/overlap  Material |
| Suturing of perineum | Number of sutures/several sutures/continuous  Suture material |
| Suturing of perineal skin | Intracutaneous/not sutured/number of sutures/sutured by midwife |
